# Editorial: The role of tumor microenvironment in malignant progression and target validation

**DOI:** 10.3389/fphar.2025.1656765

**Published:** 2025-09-25

**Authors:** Xu Zhang, Xiyue Zhang, Weifeng Zhang, Bo Kong, Runbin Sun, Qianming Du

**Affiliations:** ^1^ Department of Pharmacy, Chengdu Integrated TCM & Western Medicine Hospital, Chengdu University of TCM, Chengdu, China; ^2^ School of Basic Medicine and Clinical Pharmacy, China Pharmaceutical University, Nanjing, China; ^3^ Changzhou LESUN Pharmaceuticals Ltd., Jiangsu, China; ^4^ Department of Pharmacology and Toxicology, Ernest Mario School of Pharmacy, Rutgers, The State University of New Jersey, Piscataway, NJ, United States; ^5^ Phase I Clinical Trials Unit, Nanjing Drum Tower Hospital, Affiliated Hospital of Medical School, Nanjing University, Nanjing, China; ^6^ Nanjing Drum Tower Hospital, Clinical College of Nanjing, University of Chinese Medicine, Nanjing, China; ^7^ General Clinical Research Center, Nanjing First Hospital, Nanjing Medical University, Nanjing, China

**Keywords:** TME, action, mechanism, tumor metastasis, treatment-resistant

The tumor microenvironment (TME) constitutes a complex network that includes several types of immune cells, such as cancer-associated fibroblasts, endothelial cells, pericytes, and various other tissue-resident cell types. The TME plays a crucial role at every stage of cancer progression. Increasing evidence indicates that the TME is not merely a passive bystander but rather a dynamic orchestrator of malignancy, driving tumor initiation, progression, metastasis, and therapeutic resistance. This Research Topic, “The Role of Tumor Microenvironment in Malignant Progression and Target Validation”, summarizes cutting-edge advances in understanding TME-centric mechanisms and their translational potential. It elucidates the effects of cell crosstalk, metabolic reprogramming, and immune matrix interactions on tumor malignant progression and drug therapy in the tumor immune microenvironment from matrix, metabolism, and immune suppression perspectives. It provides new strategies to overcome drug resistance and validate different cancer targets from the TME perspective.

The complexity of the TME stems from its diverse cellular components uniquely contributing to malignancy. In this Research Topic, Lan et al. focus on a perspective article examining the multi-omics analysis of the dynamic role of STAR + cells in regulating responses to platinum-based chemotherapy and their influence on the tumor microenvironment in serous ovarian carcinoma (SOC). This study reveals that STAR + cells, identified as a novel subtype of cancer-associated fibroblasts, are key regulatory factors in the chemotherapy response of SOC. By modulating Wnt signaling and metabolic pathways (such as P450/lipid metabolism), these cells enhance platinum sensitivity and predict patient survival, positioning STAR + as both a biomarker and co-target for overcoming resistance. In addition, Tumor-associated macrophages (TAMs) in gastric cancer orchestrate immune suppression, therapy resistance, and angiogenesis. Wan et al. reviewed the targeting of TAMs in gastric cancer progression and therapy, focusing on molecular mechanisms and therapeutic applications. Targeting TAMs via depletion (e.g., photodynamic therapy), repolarization (e.g., PI3Kγ inhibitors), or CAR-M strategies restores antitumor immunity and synergizes with chemotherapy. Lymphatic metastasis is the result of the cooperative effects of tumor cells, the TME, and pre-metastatic niches. Wang et al. reviewed the impact of the dynamic and complex interplay between T cells, B cells, TAMs, neutrophils, and cancer-associated fibroblasts of the TME on lymphatic metastasis, highlighting novel opportunities for therapeutic targeting of the TME. Collectively, these studies underscore that the spatial positioning and functional plasticity of TME cells dictate metastatic fitness, providing a new direction for exploring the role of the TME in the malignant progression of tumors.

Metabolic adaptations within the TME fuel progression and evade therapies. Metabolic Reprogramming has become an important research field focused on therapeutic resistance. Key findings include: Hypoxia-induced tRNA fragments (tRFs), exemplified by tRF-3Thr-CGT, rewire hepatocellular carcinoma metabolism. By inhibiting mitochondrial peptide deformylase (HsPDF), tRFs suppress oxidative phosphorylation (OXPHOS), amplify glycolysis, and enhance invasion—vulnerabilities that tRF inhibitors exploit to reduce tumor growth *in vivo* (Qu et al.). Sun et al. investigated thepesticide-driven Warburg effect, revealing that chronic exposure to fenvalerate activates ROS-AKT/AMPK signaling, thereby enhancing glycolytic flux in liver cancer. This metabolic rewiring induces resistance to metformin, highlighting environmental toxins as covert accelerants of TME-driven therapeutic escape. These studies reveal that metabolic crosstalk between tumor and stromal cells creates a permissive niche for aggressive behavior and resistance.

Metabolic reprogramming serves as a mechanism of treatment resistance by which microenvironmental stressors induce metabolic adaptations in both tumor and immune cells, ultimately creating refractory niches. Recent mechanistic advances particularly elucidate how TME-rewired metabolic circuits fuel therapeutic resistance. Cholesterol accumulation within the TME induces exhaustion in CAR-NK cells by disrupting lipid rafts and inhibiting cytokine production, suggesting that targeting cholesterol metabolism—through inhibitors such as statins or LXR agonists—can rejuvenate immune cell function and enhance the efficacy of CAR-based immunotherapies (Liu). In addition, Rassouli et al. emphasize hypoxia-driven metastasis via HIF-1α, where sunitinib and its analogs inhibit HIF-1α dimerization, reducing EMT, migration, and invasion in colorectal cancer models, validating HIF-1α as a druggable target for anti-metastatic therapy. Collectively, these findings underscore the TME’s influence on cancer progression and validate metabolic (e.g., cholesterol pathways) and molecular (e.g., HIF-1α) targets for combinatorial therapies, offering promising strategies to overcome resistance and improve clinical outcomes in solid tumors.

**FIGURE 1:**
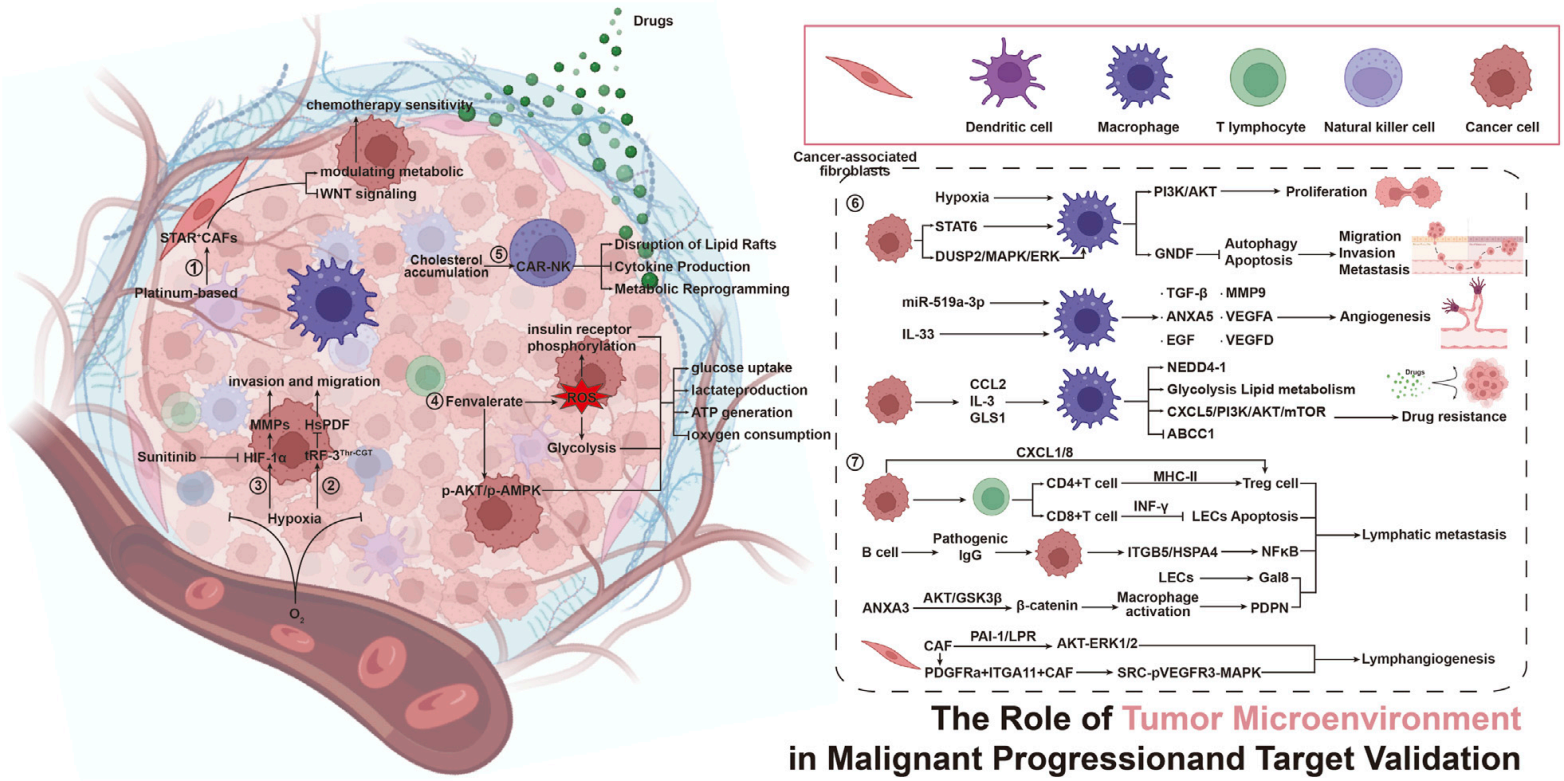
The role of tumor microenvironment in malignant progression and target validation.

In summary, this Research Topic crystallizes a paradigm shift: the TME is both a barrier and a gateway to curing cancer. By dissecting its metabolic, immune, and stromal axes, these studies provide a roadmap for rational target validation and combination therapy design (Figure 1). We extend our gratitude to the authors, reviewers, and Frontiers in Pharmacology editorial team for their invaluable contributions to advancing this frontier.

1. Multi-omics analysis of the dynamic role of STAR + cells in regulating platinum-based chemotherapy responses and tumor microenvironment in serous ovarian carcinoma (Figure 1, Action ①)
2. Hypoxia-induced tRF-3^Thr−CGT^ promotes hepatocellular carcinoma progression via mitochondrial energy metabolism remodeling dependent on the mtDNA-translation mechanism (Figure 1, Action ②)
3. Exploring the anti-metastatic potential of sunitinib and novel analogs in colorectal cancer: insights into HIF-1α mediated metastasis (Figure 1, Action ③)
4. Fenvalerate exposure induces AKT/AMPK-dependent alterations in glucose metabolism in hepatoma cells (Figure 1, Action ④)
5. Cholesterol metabolism: a positive target to revoke the function of exhausted CAR-NK cells in tumor microenvironment (Figure 1, Action ⑤)
6. Targeting tumor-associated macrophages in gastric cancer progression and therapy: insights from molecular mechanisms to therapeutic applications (Figure 1, Action ⑥)
7. Cellular components of tumor microenvironment: understanding their role in lymphatic metastasis of tumors (Figure 1, Action ⑦).

